# A Reference Assembly for the Legume Cover Crop Smooth Vetch *Vicia villosa* Roth var. *glabrescens*

**DOI:** 10.3390/biology15050379

**Published:** 2026-02-26

**Authors:** Zhongxu Yao, Xinru Li, Yurou Wang, Yaqi Sun, Songchong Lu, Kunlong Su, Huajie Zhang, Shaoyong Yang, Guofeng Yang, Lichao Ma

**Affiliations:** 1 College of Grassland Science, Qingdao Agricultural University, Qingdao 266109, China; 17660433151@163.com (Z.Y.); lixinru0404@163.com (X.L.); 15092927167@163.com (Y.W.); 19506112924@163.com (Y.S.); lusch@qau.edu.cn (S.L.); sukl@qau.edu.cn (K.S.); 2 Key Laboratory of National Forestry and Grassland Administration on Grassland Resources and Ecology in the Yellow River Delta, Qingdao 266109, China; 3 Weihai Animal Epidemic Disease Prevention and Control Center, Weihai 264299, China; zhanghuajie89@163.com; 4 Weihai Academy of Agricultural Sciences, Weihai 264299, China; 5 Bureau of Marine Development and Fisheries, Hekou District, Dongying 257299, China; zxygglz@163.com; 6 Agricultural Research Institute of Saline and Alkaline Land of Yellow River Delta, Dongying 257000, China

**Keywords:** *Vicia villosa*, genome assembly, heterozygosity, transposable elements, gene family evolution

## Abstract

*Vicia villosa* Roth var. *glabrescens* (smooth vetch) is an economically important legume cover crop valued for its nitrogen-fixing capacity, high biomass yield, and adaptability across diverse agroecosystems. Here, we present a chromosome-scale, high-quality genome assembly of *V. villosa* var. *glabrescens*, constructed using PacBio HiFi sequencing combined with Hi-C scaffolding. The assembly spans 3.70 Gb with a scaffold N50 of 4.69 Mb and exhibits lower heterozygosity (0.9%) compared to *V. villosa* Roth (3.1%). Genome analysis revealed significant expansion of long terminal repeat retrotransposons (LTR-RTs), as well as lineage-specific proliferation of miniature inverted-repeat transposable elements (MITEs) in *V. villosa* var. *glabrescens.* Comparative genomics with *V. villosa* Roth highlighted gene family expansions associated with trichome development, providing insights into the genetic basis of morphological and adaptive differences within the *Vicia* species. This reference genome provides a foundational resource for accelerating the breeding of *V. villosa* varieties with enhanced agronomic traits and contributes to a broader understanding of legume genomics and plant genome evolution.

## Simple Summary

This study has, for the first time, completed the chromosome-level genome assembly of smooth wild pea (*Vicia villosa* var. *glabrescens*). This variety is an important leguminous cover crop and has attracted much attention due to its strong nitrogen fixation ability, high biomass, and wide adaptability. By integrating PacBio HiFi long-read sequencing and Hi-C chromatin conformation capture technology, a high-quality genome of 3.70 Gb in size was constructed. This genome has good continuity (with a scaffold N50 of 4.69 Mb), and high completeness (with a BUSCO completeness of 96.05%), and most of the sequences were successfully anchored to seven pseudo-chromosomes. This reference genome (named Vvill-gla1.0) provides an important resource for in-depth analysis of key agronomic traits of Vicia gladiata (such as seed dormancy and stress resistance), will strongly promote its molecular breeding process, and enhance the understanding of genome evolution in leguminous plants.

## 1. Introduction

*Vicia villosa* var. *glabrescens* is a winter-hardy annual legume, widely recognized as a valuable cover crop owing to its nitrogen-fixing capacity, high biomass production, and adaptability across diverse agroecosystems [1]. Native to Europe and Western Asia, it represents an incompletely domesticated member of the Fabaceae family. As a key component in sustainable agriculture, it improves soil fertility, suppresses weeds, and reduces erosion [2]. Despite these agronomic benefits, its broader adoption is hindered by two persistent domestication barriers: seed dormancy and pod dehiscence, traits largely eliminated in modern crops through millennia of selection. The genus *Vicia* comprises several agriculturally important species utilized as cover crops and forage, with *Vicia villosa* Roth (hairy vetch) being widely cultivated for its winter hardiness and nitrogen-fixing capacity [3]. Recent genome-wide association studies (GWAS) in *V. villosa* have identified a major quantitative trait locus (QTL) controlling seed dormancy; however, the absence of a high-quality reference genome has hindered functional characterization of candidate genes and constrained marker-assisted breeding efforts [4].

Genomic resources have accelerated trait dissection in leguminous forages such as alfalfa (*Medicago sativa*) and *Vicia sativa*, accelerating the dissection of domestication traits [5,6]. The *V. villosa* Roth reference genome (V.vill1.0) has recently been published, revealing a highly heterozygous (3.1%) and repetitive (81.1%) genome, reflecting its predominantly outcrossing reproductive strategy [3,7]. Genetic variation within this species has been associated with traits that reduce weediness and improve biomass quality [8]. In *Vicia sativa* ssp. *amphicarpa*, an amphicarpic relative that was recently sequenced, terpenoid biosynthesis in subterranean pods was linked to antimicrobial resistance, illustrating how genomic insights can elucidate adaptive mechanisms [5]. On the other hand, *V. villosa* var. *glabrescens* remains genomically underexplored, with prior research relying on fragmented assemblies that preclude comprehensive evolutionary and functional analyses. Persistent seed dormancy in *V. villosa* results in volunteer plants that compete with cash crops, while pod dehiscence contributes to substantial seed yield losses—issues exacerbated in mechanized farming systems. Traditional breeding strategies, though partially effective, are labor-intensive and slow due to the polygenic architecture of these traits. A chromosome-level genome assembly would enable precise identification of causal variants, comparative genomics analyses to trace domestication signatures, and the development of molecular markers to accelerate trait introgression.

Here, we report the first chromosome-scale genome assembly of *V. villosa* var. *glabrescens*, generated using PacBio HiFi sequencing and Hi-C scaffolding. The assembly comprises 3.70 Gb and includes 58,515 annotated protein-coding genes. We identified substantial expansion of long terminal repeat retrotransposons (LTR-RTs), contributing to the genome’s large size. This assembly provides a critical foundation for understanding the genomic basis of morphological and adaptive differences within the Vicia species and provides an essential resource for breeding improved cover crop varieties with enhanced agronomic performance.

## 2. Methods

### 2.1. Sample Selection and Sequencing

A DNA secure kit (TianGen, Beijing, China) was used to isolate genomic DNA from the leaves of *V. villosa* var. *glabrescens* in the Grassland Agri-Husbandry Research Center, College of Grassland Science, Qingdao University, and its quality was assessed with a Nano Photometer spectrophotometer [9]. High-quality DNA was used for sequencing. To mitigate challenges posed by high heterozygosity and repetitive sequences, a multi-platform sequencing strategy was adopted. Illumina sequencing was employed to generate short-read data for high accuracy and coverage, while PacBio single-molecule real-time (SMRT) sequencing provided long reads to resolve repetitive regions. Specifically, a paired-end library with a 300–350 bp insert size was constructed and sequenced on the Illumina PE150 platform. PacBio SMRT libraries of 10 kb were prepared and sequenced using the PacBio Sequel II platform. Additionally, Hi-C technology [10] was applied to facilitate chromosome-level scaffolding by providing long-range linkage information to improve assembly contiguity and accuracy.

Genomic DNA was extracted using a modified CTAB protocol [9], with additional purification steps including chloroform-isoamyl alcohol extraction and ethanol precipitation, followed by cleanup using silica-column-based kits (Regenerated Silica Columns, as described in Fu et al. [9], to remove polysaccharides, polyphenols, and other inhibitors.

All PacBio subreads were processed into circular consensus sequences (CCS) with a minimum pass count = 3, resulting in high-fidelity HiFi reads suitable for de novo assembly

### 2.2. Genome Assembly and Annotation

**Figure 1:**
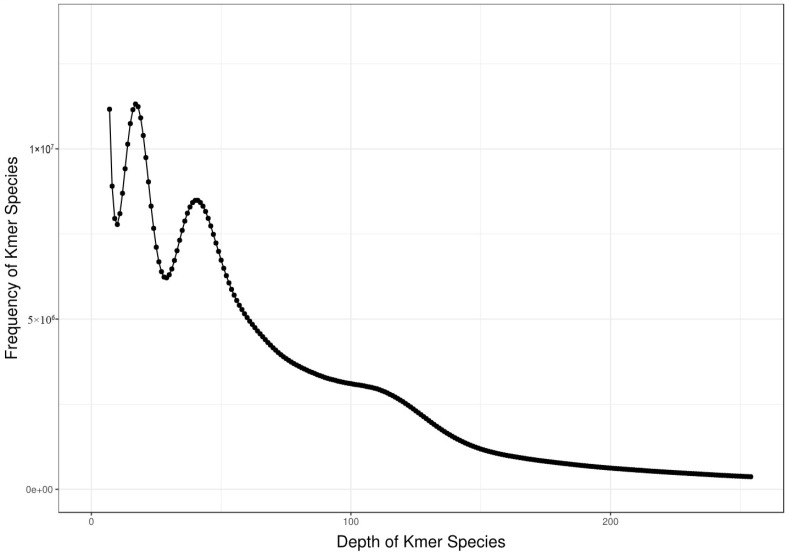
K-mer frequency distribution (K-mer = 17) from Illumina short reads, a technical profile used for preliminary estimation of genome size, heterozygosity, and repeat content prior to genome assembly. Note: This distribution reflects the frequency of short-read k-mers and provides an initial technical reference, rather than a direct biological description of the genome.

Genome survey analysis was performed to estimate the genome characteristics prior to de novo assembly. Briefly, high-quality Illumina short reads were subjected to k-mer counting using Jellyfish (v2.3.0) [11]. The obtained k-mer frequency histogram (Figure 1) was then analyzed with GenomeScope 2.0 to estimate the genome size, heterozygosity rate, and repeat content. This k-mer-based approach is a standard practice in modern plant genome projects [12].

The k-mer analysis performed in this study is a technical tool for preliminary genome survey, rather than a definitive method for describing genome biology. While our k-mer-based estimates provided a useful starting point for genome assembly, these values are subject to potential biases from sequencing errors and complex repeat sequences.

We chose Khaper for haplotype purging due to its compatibility with HiFiASM outputs and its demonstrated efficacy in reducing false duplications in highly repetitive plant genomes. While tools like purge_dups are widely used, they are primarily optimized for error-prone long reads (e.g., ONT), whereas Khaper leverages the high accuracy of HiFi data to distinguish true duplications from allelic variants. The following default parameters were used: –min_cov 5, –max_cov 80, and –block_len 5000. Post-purge BUSCO analysis showed a marked reduction in duplicated genes compared to the raw assembly.

The LTR_harvest and LTR_FINDER_parallel software were used to predict total LTRs across the genome. Subsequently, the LTR_retriever software was employed to remove false positives, yielding a high-quality LTR-RTs library. The long terminal repeat sequences at both ends of LTR-RTs are identical at the initial moment of transposition. Over time, both LTR sequences accumulate mutations, leading to sequence divergence. Based on molecular clock theory, by calculating the divergence between the two LTRs and using the mutation rate of closely related species as a reference, the insertion time of the transposon can be estimated. Studying LTR burst times provides insights into species evolution and related topics.

Genome size and heterozygosity of *V. villosa* var. *glabrescens* were estimated using k-mer frequency distribution analysis via Jellyfish and GenomeScope. The PacBio SMRT reads were assembled using HiFiASM (0.20.0 r639), and potential duplicate haplotypes were removed using Khaper 2. Subsequently, the Hi-C data were mapped to the assembled contigs using BWA (0.7.12-r1039), and reads with unique mapping positions were retained. The resulting data were subsequently scaffolded into seven pseudochromosomes using Juicer (1.6) and 3D-DNA [13]. Genome assembly completeness was evaluated using BUSCO (5.3.1) [14], Merqury [15], and LTR Assembly Index [16]. For repeat element annotation, the EDTA pipeline was utilized, integrating multiple TE annotation tools such as RepeatModeler, LTR_FINDER, LTRharvest, and LTR_retriever.

Both eukaryotes and prokaryotes rely on genes, which store information for nearly all life processes, including growth, development, and apoptosis. Non-coding RNA does not encode proteins but plays crucial regulatory roles in various biological processes, including development, proliferation, transcription, post-transcription modification, apoptosis, and cellular metabolism. Repeat sequences can jump or replicate within the genome, altering its sequence and thereby influencing evolution, inheritance, and variation. They are indispensable for gene expression, transcriptional regulation, chromosome architecture, and physiological metabolism.

Protein-coding genes in the genome of *V. villosa* Roth var. *glabrescens* were predicted using a combined approach integrating de novo, homology-based, and transcriptome-based approaches. Gene models were first generated with BRAKER, leveraging RNA-seq data to enhance prediction accuracy. This model was subsequently utilized for ab initio prediction within AUGUSTUS. For homology-based prediction, protein sequences from 12 plant species, including *V. villosa* Roth, *Glycine max*, *Lotus japonicus*, *Medicago sativa*, *Medicago truncatula*, *Pisum sativum*, *Vigna radiata*, *Trifolium subterraneum*, *Trifolium pratense*, *Arabidopsis thaliana*, and *Cicer arietinum*, were aligned to the *V. villosa* var. *glabrescens* genome using TBLASTN, with an E-value threshold set at 1 × 10^−5^ (https://blast.ncbi.nlm.nih.gov/Blast.cgi, accessed on 1 October 2024). Homologous genes were further scrutinized using Genewise and GeMoMa [17] to refine predictions. Transcriptome-based prediction was conducted using PASA and TransDecoder, incorporating RNA-seq data from mixed samples to enrich the prediction dataset. Ultimately, Maker was deployed to amalgamate all prediction outcomes, yielding a unified and reliable gene set.

Protein-coding genes refer to genes capable of encoding proteins. Composed of hundreds to thousands of nucleotide sequences, they are transcribed and translated to encode specific proteins. Their coding sequences determine the sequence of the encoded protein, thereby dictating the protein’s structure and function.

Comprehensive studies of protein-coding genes play a vital role in comparative genomics research, understanding biological processes and regulation within organisms, and advancing disease treatment.

Non-coding RNAs were annotated using tRNAscan-SE [18] for tRNA identification, and Infernal cmscan with the Rfam [19] database for miRNA, rRNA, and snRNA detection. Functional annotation of the predicted protein-coding genes was achieved through BLASTP, referencing publicly accessible databases such as Nr, KEGG, SwissProt, and InterProScan. Gene Ontology (GO) terms were assigned using EggNOG to provide a broader functional categorization of the identified genes.

### 2.3. Comparative Genomic Analysis

Orthologous protein groups were identified using OrthoFinder [20] across *V. villosa* Roth var. *glabrescens* and the related species. For the detection of expanded and contracted gene families, single-copy gene families were concatenated and aligned using OrthoMCL [21]. A maximum likelihood phylogenetic tree was subsequently constructed with RAxML [22]. Divergence times were estimated by calibrating the tree with fossil-based records from the TimeTree database (http://www.timetree.org/).

Genome collinearity analysis was performed using MCScanX and visualized through JCVI [23]. The non-synonymous (Ka) and synonymous (Ks) substitution rates for gene pairs were calculated using the CodeML model in KaKs_Calculator [24]. Finally, GO and KEGG enrichment analyses were conducted using the clusterProfiler package v3.22.

## 3. Results

### 3.1. Genome Sequencing, Size Estimation, and Assembly

In this study, we characterized the genome of *Vicia villosa* var. *glabrescens* through integrated genomic approaches. Whole-genome sequencing was performed using a combination of Illumina short-read, PacBio SMRT long-read sequencing, and Hi-C scaffolding. To obtain preliminary genomic characteristics, we first conducted a genome survey using Illumina data: 193.48 Gb of high-quality paired-end reads were generated (Table 1) and subjected to k-mer analysis (k = 17), a technical method that enabled us to estimate the genome size at ~4697 Mb, heterozygosity at 0.90%, and repeat content at ~90.13% (Figure 1). These k-mer-based estimates served as a foundational reference for subsequent genome assembly and validation.

Fluorescence in situ hybridization of telomeric regions confirmed a diploid chromosome number of 2*n* = 14. De novo assembly using PacBio Circular Consensus Sequencing (CCS) data produced a 3.70 Gb genome, comprising 164 contigs with a contig N50 of 4.93 Mb (Table 2).

Based on NT library alignment results, no significant exogenous contamination was detected in the library data. K-mer analysis revealed a sample genome size of approximately 4719 Mb, adjusted to 4697 Mb, with a heterozygosity rate of 0.90% and a repetitive sequence proportion of 90.13%.

**Figure 2:**
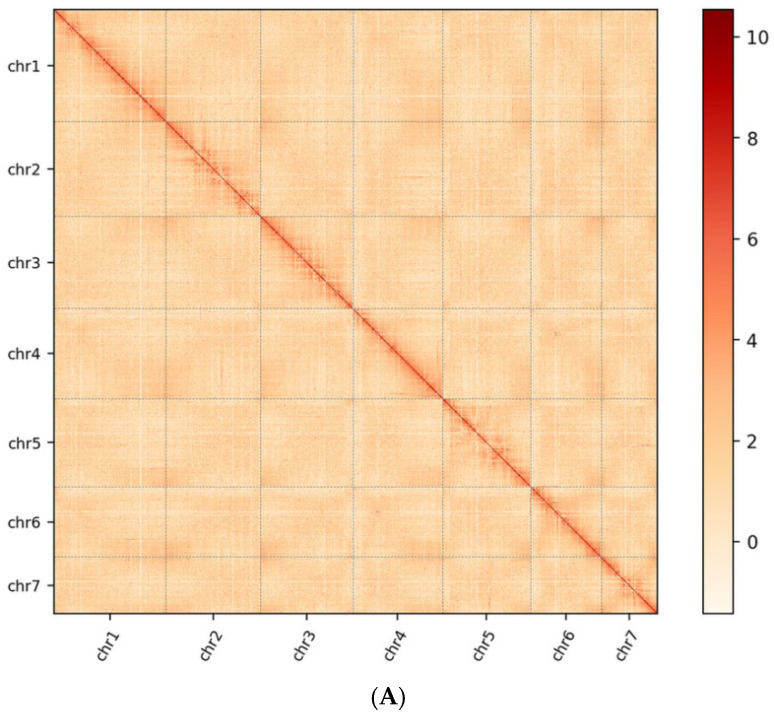

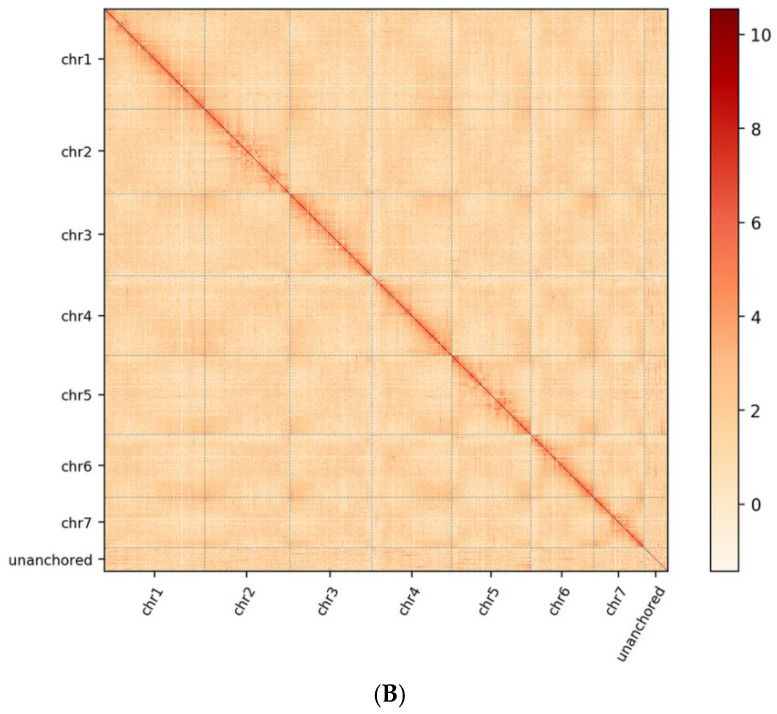
(**A**) Chromosome Hi-C interaction map; 1 Mb resolution interaction map. (**B**) Genome Hi-C interaction map; 1 Mb resolution interaction map. In the figure, the colors from light to dark represent the increase in interaction intensity. The darker the color, the stronger the interaction. The horizontal and vertical coordinates represent their N*bin positions on the genome. The first 7 squares in the figure represent the 7 chromosomes of *Vicia villosa* var. *glabresens*, and the rest are sequences that have not been clustered onto chromosomes.

During assembly correction, the original 3124 contigs were broken down based on interaction maps to identify erroneous contigs, which were then reordered. This process ultimately yielded 7 chromosomes and 383 scaffolds with a total length of 3.03 Gb, a contig N50 of 4.93 Mb, and a scaffold N50 of 431.83 Mb. The chromosome anchoring rate reached 95.79%. Subsequently, species-specific chromosome and genome-wide interaction maps were constructed, as shown in Figure 2A,B. The results align with interaction patterns, indicating successful Hi-C-assisted assembly for this species.

BUSCO analysis using the embryophyta_odb10 dataset revealed that 96.05% of complete BUSCO genes could be detected in the assembly, indicating a high level of genome completeness (Table 3).

### 3.2. Assembly Annotation Evaluation

**Figure 3:**
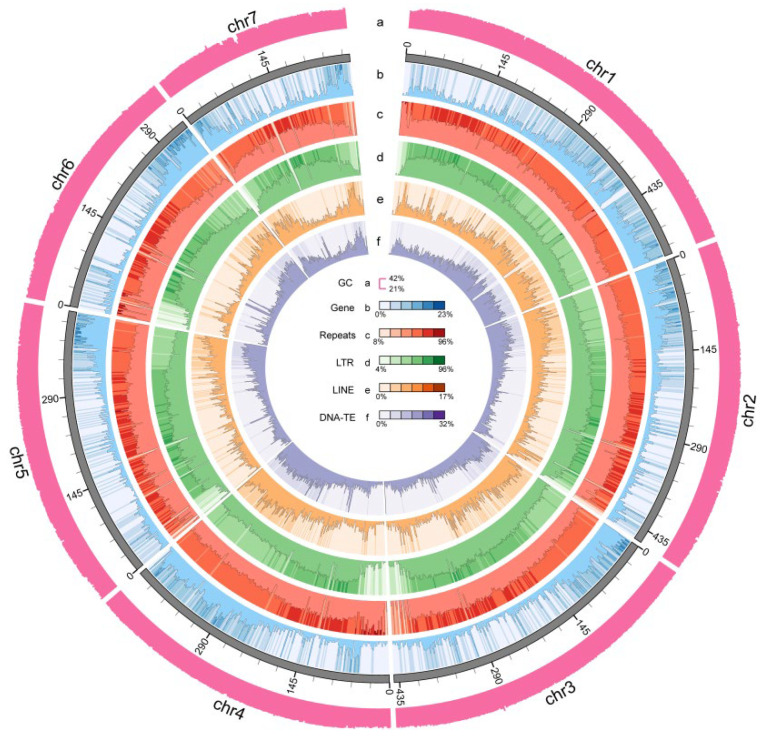
Overview of *Vicia villosa* var. *glabrescens* genome assembly.

Repeat annotation revealed that repetitive elements constitute 63.18% of the genome (Table 4), with Long Terminal Repeat Retrotransposons (LTR-RT) being the most prevalent type, comprising approximately 51.02% of the genome (Table 5). Utilizing a combination of de novo, homology-based, and transcriptome-based approaches, a total of 58,515 protein-coding genes were identified and annotated, of which 98.98% were functionally characterized (Table 6). Additionally (Figure 3), the genome was predicted to encode 501 miRNAs, 2215 tRNAs, 3550 rRNAs, and 2695 snRNAs (Table 7).

**Figure 4:**
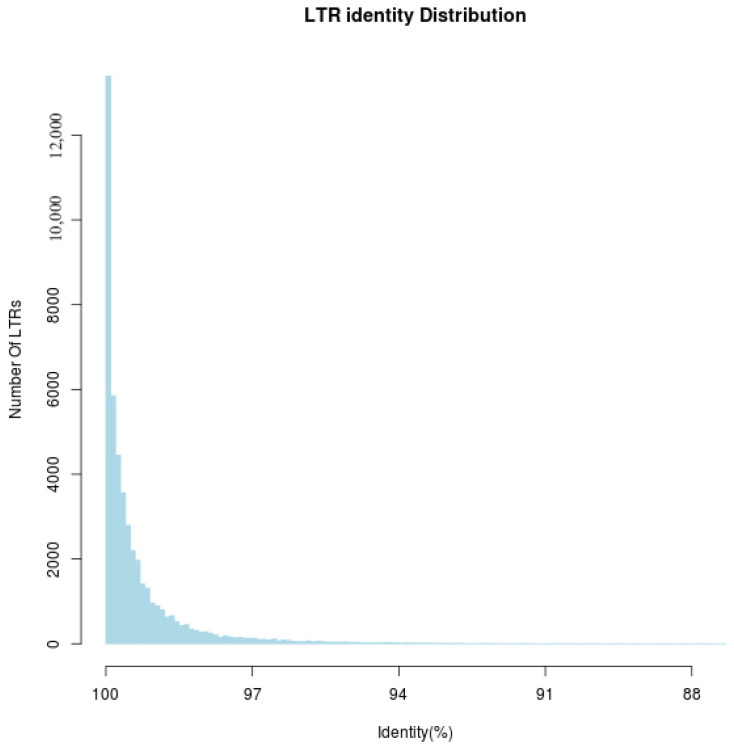
Statistical distribution of LTR terminal sequence consistency. Horizontal axis: represents the percentage of identity (identity (%)). Vertical axis: represents the number of LTRs (number of LTRs).

As shown in the figure, the number of LTRs (Figure 4) peaks at 100% consistency and then rapidly decreases as the consistency percentage declines. Most LTRs are concentrated in high-consistency regions, while low-consistency regions contain fewer LTRs. The overall distribution exhibits a pronounced right skew.

Based on evidence-based prediction results (Table 8 and Table 9), a total of 150,719 protein-coding genes were identified. These gene models are unevenly distributed across the seven chromosomes. The average gene length is 3528 bp, with each gene containing an average of four exons. The average lengths of the coding sequence (CDS), exons, and introns are 1345 bp, 335.98 bp, and 726.79 bp, respectively. Using the software liftoff to analyze homologous proteins from related species yielded a total of 48,311 protein-coding genes. These gene models are unevenly distributed across the seven chromosomes. The average gene length is 2394 bp, with each gene containing an average of 4.22 exons. The average lengths of the coding sequence (CDS), exons, and introns were 1232 bp, 292.29 bp, and 361.43 bp, respectively.

### 3.3. Comparative Genomic and Phylogenomic Analyses

**Figure 5:**
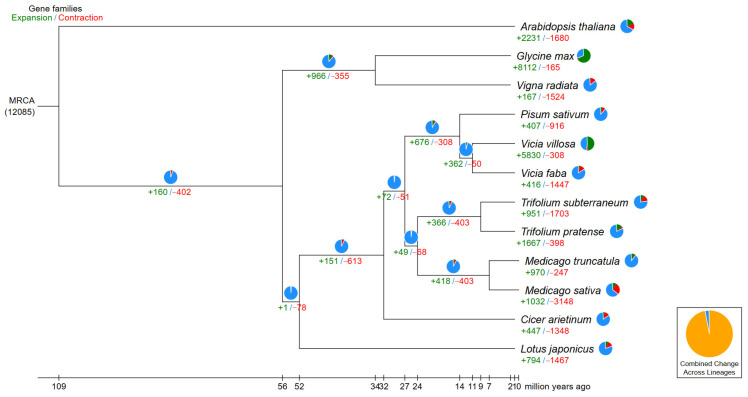
Gene family expansion and contraction diagram notice: As shown in the figure above, green numbers indicate the number of gene families that underwent expansion during species evolution, while red numbers indicate the number of gene families that underwent contraction.

The *V. villosa* var. *glabrescens* genome was compared with those of 11 legume plant species (*Glycine max*, *Vigna radiata*, *Pisum sativum*, *Vicia faba*, *Phaseolus vulgaris*, *Trifolium subterraneum*, *Trifolium pratense*, *Medicago truncatula*, *Medicago sativa*, *Cicer arietinum*, *Lotus japonicus*) and the outgroup *Arabidopsis thaliana*. Phylogenetic analysis of single-copy orthologs confirmed the close evolutionary relationship between *Vicia villosa* and *Vicia villosa* var. *glabrescens* (Figure 5), with an estimated divergence time of approximately 9.6 million years ago (MYA) (Figure 5).

**Figure 6:**
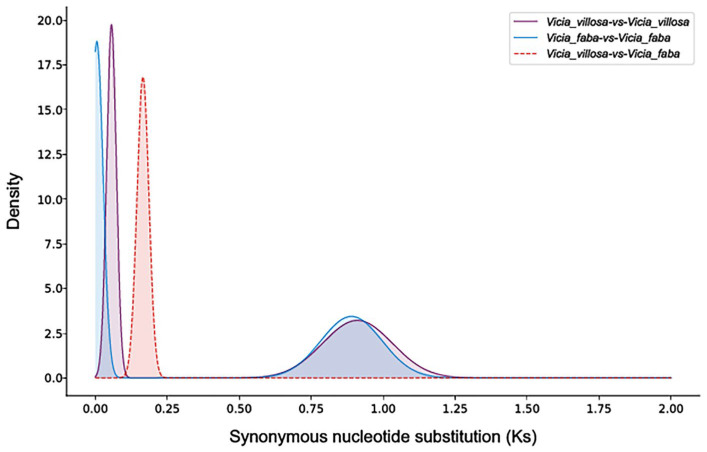
Multi-species Ks distribution plot. Peaks in interspecies combination plots represent species divergence, while peaks in intraspecies combination plots indicate whole-genome duplication events within the same species.

**Figure 7:**
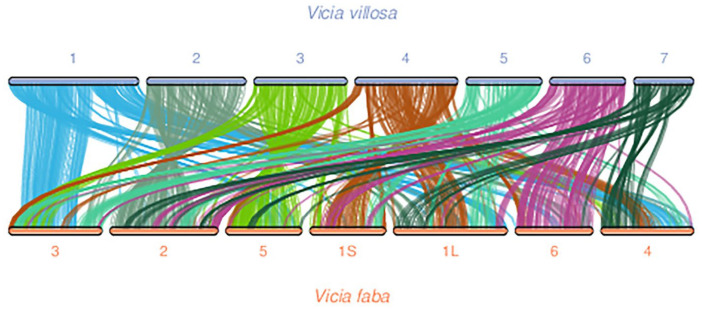
Collinearity diagram of genes encoded by two species. Note: (1). Species vs Species: One species vs another species; (2) Syntenic Blocks: Number of syntenic blocks; (3) Average Syntenic Gene Pairs Per Block: Average number of syntenic gene pairs per syntenic block; (4) Gene Pairs Included in Syntenic Block: Total number of syntenic gene pairs contained within a syntenic block; (5) Median Block Length: Median length of syntenic blocks.

A whole-genome duplication (WGD) analysis using WGDI was performed to compare the genomes of *V. villosa* var. *glabrescens* (*n* = 7) and *V. villosa* (*n* = 6). Ks values derived from collinear genes were used to generate a frequency distribution plot. The overlapping peak P2 indicates that both species underwent a common ancestral WGD event prior to their divergence (Figure 6). Collinearity was also detected between *V. villosa* var. *glabrescens* and *V. villosa* as well as *V. faba* (Figure 7). One-to-one chromosome pairing was observed between *V. villosa* var. *glabrescens* and *V. villosa*. However, some chromosomes in *V. villosa* var. *glabrescens* are paired with multiple chromosomes in *V. villosa*. Additionally, certain chromosome fragments in *Vicia villosa* var. *glabrescens* aligned to more than one chromosome in *V. faba* (Figure 7). These findings suggest that after a common WGD event in these species, chromosomal rearrangements occurred, resulting in the deletion of paralogous genes.

### 3.4. Gene Family and Evolution Analysis

**Figure 8:**
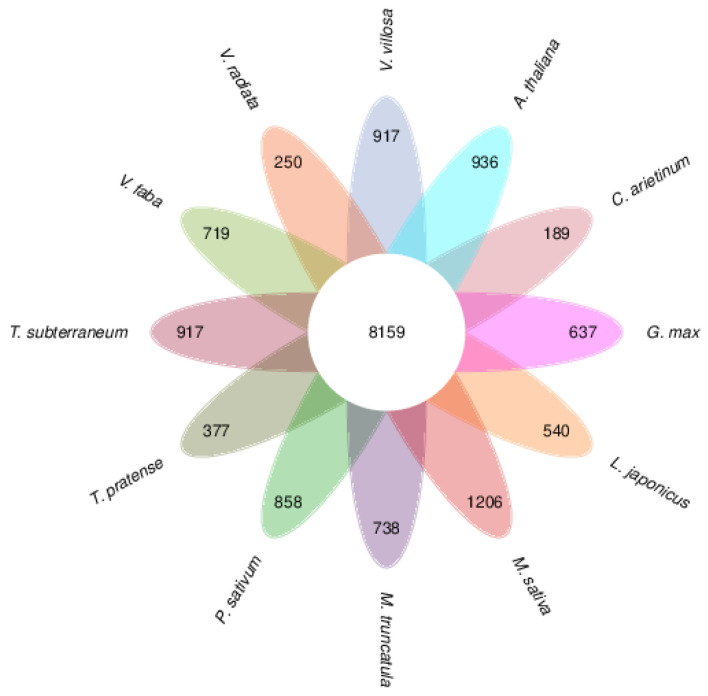
Gene family clustering petal diagram. Overlapping areas between circles represent gene families shared among all species. Numbers indicate the number of gene families, with surrounding circles representing each species.

**Figure 9:**
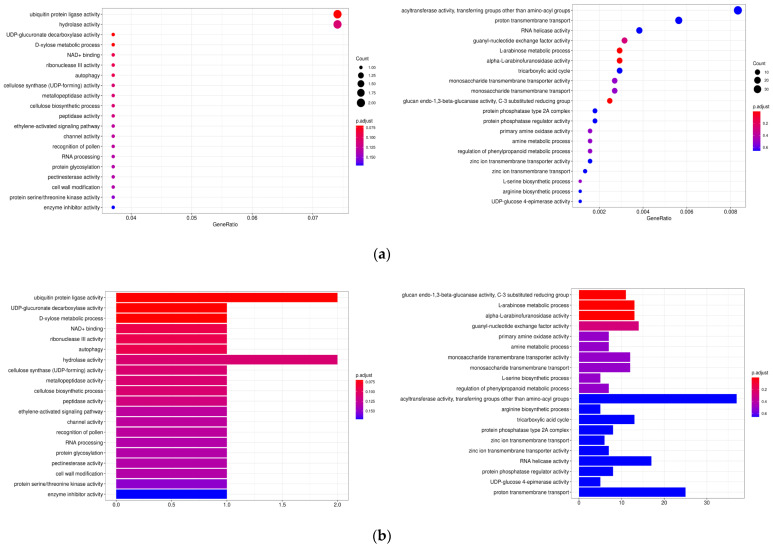
Functional enrichment of the expanded gene families in *V. sativa* ssp. amphicarpa. (**a**) The GO and (**b**) KEGG enrichment analysis of the expanded gene families.

Gene family clustering grouped 56,772 *V. villosa* var. *glabrescens* genes into 18,918 families, with 8159 families shared across all species analyzed (Figure 8). Evolutionary analysis revealed that *V. villosa* var. *glabrescens* experienced expansion in 5830 gene families and contraction in 308 gene families during its evolutionary history. Functional enrichment GO analysis of expanded families revealed significant overrepresentation of pathways associated with flavonoid biosynthesis (Figure 9).

The relatively low heterozygosity (0.9%) may suggest a shift toward increased self-fertilization, although direct reproductive studies are needed to confirm mating system dynamics in this population, potentially involving orthologs of known regulators such as GLABROUS1, though functional validation is required to establish causality. The distinct MITE proliferation patterns suggestive of transposon-mediated genomic flexibility, which may contribute to phenotypic diversification within the *V. villosa* complex—a hypothesis warranting further investigation.

Compared to *V. villosa* var. *glabrescens,* which exhibits markedly reduced duplication of BUSCO genes (12.3% vs. 36.8%)—indicative of a more stable genomic architecture—this aligns with its lower heterozygosity and supports its use in breeding programs towards improving genetic uniformity in cover crops.

## 4. Discussion

This study presents the first chromosome-scale genome assembly of *Vicia villosa* Roth var. *glabrescens*, providing valuable resources for understanding the genomic basis of this species and enabling targeted improvement of its agronomic traits.

The assembled genome of *Vicia villosa* var. *glabrescens* spanned 3.70 Gb and exhibited a lower heterozygosity level compared to *V. villosa* Roth (3.1%), suggesting enhanced genomic stability. Lower heterozygosity may suggest increased selfing tendency, a hypothesis that requires verification through pollination experiments, which is beneficial for establishing uniform cover crop populations. Additionally, repetitive elements constituted 63.18% of the genome of *Vicia villosa* var. *glabrescens*, with LTR-RTs being the most abundant, accounting for 51.02%. The expansion of these repetitive elements may have significantly influenced genome architecture and the evolutionary trajectory of *Vicia villosa* var. *glabrescens*.

The discrepancy between the k-mer estimated genome size (4.697 Gb) and the final assembly (3.70 Gb) likely reflects the exclusion of highly repetitive (Table 2), low-complexity, or heterochromatic regions that are difficult to assemble even with long reads. These regions often consist of tandem repeats and degenerate transposable elements that collapse during assembly. Additionally, potential over-purging of haplotypic regions during diploid-aware assembly cannot be ruled out.

Despite a low overall heterozygosity (0.9%), the BUSCO analysis revealed 61.07% duplicated genes. This apparent contradiction may reflect either incomplete haplotype purging or genuine lineage-specific gene family expansions. Given that *V. villosa* var. *glabrescens* has undergone recent whole-genome duplication events (Figure 2B), some duplication signal may represent retained paralogs rather than allelic redundancy. Nevertheless, we acknowledge that current metrics suggest room for improvement in diploid resolution, and future efforts may benefit from trio-binning or phased assembly approaches.

This study focuses on the first telomere-to-telomere human genome assembly (CHM13) completed using long-read sequencing technology, which resolves the challenges in complex regions such as segmental duplications and centromeric satellite arrays. Although the initial assembly was based on high-precision sequences, the evaluation still found minor errors and structural misassemblies. To address this, the researchers developed a novel repeat-aware polishing strategy capable of accurately correcting large repetitive sequences without over—correcting. This strategy successfully fixed 51% of the existing errors and increased the assembly quality value (QV) from 70.2 to 73.9. By comparing it with traditional automated polishing tools, the study provides practical advice for genome projects with limited resources to avoid common polishing errors [25].

Comparative genomic analysis revealed a shared whole-genome duplication (WGD) event in the evolutionary history of *V. sativa* subsp. *sativa* and subsp. *nigra*. However, during subsequent evolution, they underwent different chromosomal rearrangements and expansions and contractions of gene families. Notably, *Vicia sativa* subsp. *sativa* underwent expansions in 5830 gene families, predominantly enriched in flavonoid biosynthesis, which may underpin its morphological and adaptive differentiation. Additionally, the differential proliferation of MITEs in *Vicia sativa* subsp. *sativa* also suggests the role of transposon-driven genomic plasticity in species adaptive radiation.

As a leguminous cover crop, *Vicia villosa* var. *glabrescens* demonstrates robust nitrogen fixation ability, high biomass, and adaptability to various agricultural ecosystems. The genomic resources generated in this study will greatly facilitate the targeted breeding of *Vicia villosa* var. *glabrescens* varieties, especially for improving agronomic traits such as nitrogen use efficiency, abiotic and biotic stress tolerance, and reduced seed dormancy. Comparative transcriptome analysis, particularly focusing on the differences in trichomes between *Vicia villosa* var. *glabrescens* and *Vicia villosa* subsp. *villosa* can further reveal regulatory networks governing these traits.

Common vetch (*Vicia sativa* L.) is an important self-pollinating annual forage legume and is of interest for drought-prone regions as a good-quality animal feedstock with low input requirements [26,27]. Owing to its low cost, high nutritional value, and broad environmental adaptation, common vetch has been utilized both as a protein source in animal feed and for human consumption [28,29]. Under water-limited conditions, *V. sativa* exhibits greater growth inhibition compared to *V. narbonensis* and *V. villosa* [30]. Zhu et al. [7] performed a de novo transcriptional analysis of whole common vetch plants under drought stress, identifying drought-responsive genes that are mainly involved in plant hormone signal transduction, glycolysis/gluconeogenesis, and phenylpropanoid biosynthesis.

Under drought stress conditions, Min et al. [31] systematically presented and comparatively analyzed the global transcriptional regulation in leaves and roots of common vetch (*Vicia sativa*), revealing both inter-tissue crosstalk and distinct tissue-specific differences within drought response pathways. The findings indicate that hormone signaling, starch and sucrose metabolism, and arginine and proline metabolism represent significantly enriched key metabolic pathways. Among these, genes within the AREB/ABF-SnRK2 signaling pathway may promote starch degradation in leaves by regulating AMY3 and BAM1 activity, thereby redistributing carbon resources to roots and enhancing the plant’s drought adaptation capacity. Furthermore, heterologous expression experiments in yeast systems validated four transcription factors as candidate genes for enhancing osmotic stress tolerance. Collectively, the constructed leaf and root drought response transcriptomes significantly deepen our understanding of the above-ground and below-ground regulatory networks and their functions in the common wild pea under water-limited conditions.

Future studies may utilize the Vvill-gla1.0 assembly as a foundation to deeply explore the genomic basis of key agronomic traits in *Vicia gladiata*. Integrating functional, comparative, and evolutionary genomics can clarify *Vicia gladiata*’s adaptive strategies at the molecular level, offering a foundation for its use in sustainable agriculture. e Additionally, technologies such as genomic selection can be utilized to accelerate the genetic improvement of *Vicia gladiata* varieties to meet the diverse demands of agricultural production.

In conclusion, we present the first chromosome-level genome assembly of *Vicia villosa* var. *glabrescens*, a valuable cover crop. The expansion of LTR retrotransposons, particularly Tekay and Retand, appears to be the primary driver behind its large genome size following speciation. This genome assembly not only provides a fundamental genetic resource for exploring adaptive traits like reduced pod shattering and seed dormancy but also opens new avenues for comparative genomics within Fabaceae. Future studies leveraging this genome could accelerate the identification of key genes governing agronomically important traits, ultimately enabling the development of improved cover crop varieties that enhance the resilience and sustainability of agricultural systems.

## 5. Conclusions

The Vvill-gla1.0 assembly offers a comprehensive resource for investigating the genetic architecture of key agronomic traits in *V. villosa* varieties. Comparative analysis with *V. villosa* Roth revealed valuable insights into morphological divergence and environmental adaptation. Future studies will leverage this resource towards the improvement of cover crop resilience and nitrogen-use efficiency.

## Figures and Tables

**Table 1:** Preliminary genome characteristics of *Vicia villosa* var. *glabrescens* estimated by k-mer analysis (K-mer = 17).

K-Mer Number	Genome Size (Mb)	Revised Genome Size (Mb)	Heterozygous Ratio (%)	Repeat (%)
193,479,822,608	4719	4697	0.90	90.13

Note: Values represent technical estimates derived from k-mer frequency distribution of Illumina short reads and were used to guide subsequent genome assembly. Final genomic characteristics will be confirmed by the completely assembled genome.

**Table 2:** Summary of genome assembly based on PacBio long reads.

	Contig Length (bp)	Contig Number
N90	546,138	1204
N80	1,047,793	716
N70	1,678,043	435
N60	2,923,514	263
N50	4,686,072	164
Total length	3,703,753,257	-
Number (≥100 bp)	-	3124
Number (≥2 kb)	-	3124
Max length	44,425,350	-

**Table 3:** Genome assembly completeness was evaluated based on BUSCO.

Type	BUSCOs Num	Percentage (%)
Complete BUSCOs (C)	5154	96.05
Complete and single-copy BUSCOs (S)	1877	34.98
Complete and duplicated BUSCOs (D)	3277	61.07
Fragmented BUSCOs (F)	19	0.35
Missing BUSCOs (M)	193	3.6
Total BUSCO groups searched	5366	100

**Table 4:** Sample of *Vicia villosa* var. *glabresens*; data statistics.

Library Name	ReadNum	Raw Bases (bp)	Clean Reads Number	Q20 (%)	Q30 (%)	GC Content (%)
GYZHS_GYZHS Total	1,943,176,130	291,476,419,500	1,943,175,026	98.12	93.48	36.32
	1,943,176,130	291,476,419,500	1,943,175,026	98.12	93.48	36.32

**Table 5:** Statistics of transposable elements in the *Vicia villosa* var. *glabrescens* genome (Supplementary Material).

Type	Repbase TEs		TE Proteins		De Novo		Combined TEs	
	Length (Bp)	% in Genome	Length (Bp)	% in Genome	Length (Bp)	% in Genome	Length (Bp)	% in Genome
DNA	86,445,661	2.85	22,253,804	0.73	75,232,541	2.48	136,332,404	4.5
LINE	48,578,882	1.6	35,879,125	1.18	60,341,012	1.99	79,559,306	2.62
SINE	170,611	0.01	0	0	17,889	0	176,200	0.01
LTR	608,187,930	20.06	458,791,581	15.13	1,104,770,503	36.43	1,547,077,613	51.02
Satellite	99,297,992	3.27	0	0	2,156,075	0.07	101,071,976	3.33
Simple_repeat	0	0	0	0	0	0	0	0
Other	59,696	0	0	0	0	0	59,696	0
Unknown	768,719	0.03	690	0	90,268,708	2.98	91,029,498	3
Total	834,479,939	27.52	516,904,726	17.05	1,319,021,309	43.5	1,915,880,955	63.18

**Table 6:** Functional annotation of protein-coding genes in *Vicia villosa* var. *glabrescens* (Supplementary Material).

Database	Number	Percent (%)
NR	57,765	98.72
SwissProt	36,500	62.38
TrEMBL	57,110	97.6
KOG	44,939	76.8
TF	3640	6.22
InterPro	50,837	86.88
GO	36,524	62.42
KEGG_ALL	56,416	96.41
KEGG_KO	20,215	34.55
Pfam	46,283	79.1
Unannotated	596	1.02
Total	58,515	100

**Table 7:** Types of non-coding RNA detected from the *Vicia villosa* var. *glabrescens* genome.

Type		Copy	Total Length (bp)	% of Genome
miRNA		501	67,495	0.002226
tRNA		2215	166,684	0.005497
rRNA	rRNA	3550	557,271	0.018378
	18S	79	138,593	0.00457
	28S	75	14,852	0.00049
	5.8S	80	12,421	0.00041
	5S	3316	391,405	0.012908
snRNA	snRNA	2695	296,471	0.009777
	CD-box	2312	241,756	0.007973
	HACA-box	114	14,857	0.00049
	splicing	269	39,858	0.001314
	scaRNA	0	0	0
Total		8961	1,087,921	0.035878

Type

Copy

Total Length (bp)

% of Genome

**Table 8:** Evidence statistics for the final gene set.

	≥20% Overlap		≥50% Overlap		≥80% Overlap	
	No.	Ratio (%)	No.	Ratio (%)	No.	Ratio (%)
P (single)	1	0	9	0.02	640	1.07
P (more)	15	0.03	19	0.03	386	0.65
H (single)	26,426.52	4.42	3648	6.11	5573	9.33
H (more)	3897	6.52	5067	8.48	8485	14.2
C (single)	0	0	0	0	0	0
C (more)	0	0	0	0	0	0
PH	53,187	89.02	50,995	85.36	44,587	74.63
PC	0	0	0	0	0	0
HC	0	0	0	0	0	0
PHC	0	0	0	0	0	0

**Table 9:** Protein-coding gene annotation results.

	Gene Set	Number	Average Gene Length (bp)	Average CDS Length (bp)	Average Exon per Gene	Average Exon Length (bp)	Average Intron Length (bp)
De novo	*GlimmmerHMM*	312,645	8738.12	2316.68	3.44	673.4	2631.42
	*AUGUSTUS*	223,558	1771.96	831.32	3.41	243.92	390.61
Homolog	*Trifolium_pratense*	82,903	5160.86	1461.57	5.23	279.31	873.97
	*Lathyrus_sativus*	69,373	2852.16	1084.31	4.03	268.78	582.63
	*Vicia_faba*	62,375	3460.79	1132.76	4.69	241.36	630.34
	*Pisum_sativum*	116,570	5055.66	1234.26	3.82	323.09	1355.05
	*Vicia_villosa*	150,719	3528.24	1345.18	4	335.98	726.79
Liftoff	*Vicia_faba*	28,046	2365.56	1126.83	4.75	237.25	330.36
	*Vicia_villosa*	48,311	2393.99	1232	4.22	292.29	361.43
BUSCO		1716	5591.47	1670.23	9.87	169.25	442.16
MAKER		61,763	3573.23	1168.28	4.16	293.62	745.65
HiFAP		59,744	3224.58	1282.74	4.54	288.09	540.56

## Data Availability

Data are contained within the article and Supplementary Materials.

## Supplementary Materials

The following supporting information can be downloaded at: https://www.mdpi.com/article/10.3390/biology15050379/s1, Figure S1: InterPro/GO/KEGG_KO/Swiss-Prot/NR Annotation Results Venn Diagram; Figure S2: GO Category Statistics Bar Chart; Figure S3: KEGG Classification Statistics Bar Chart; Figure S4: Distribution of Divergence Levels Among Four Transposable Element Sequences Annotated by RepeatMasker; Figure S5: Distribution of divergence levels among four types of transposable element sequences predicted by de novo method.
