# Supplementary material for: A Reference Assembly for the Legume Cover Crop Smooth Vetch Vicia villosa Roth var. glabrescens
Source: Biology (Basel). 2026 Feb 26;15(5):379. doi: 10.3390/biology15050379 (PMC12984296; doi:10.3390/biology15050379)
Supplement: Supplementary file 1 [file biology-15-00379-s001.zip › biology-4027610-supplementary.pdf]

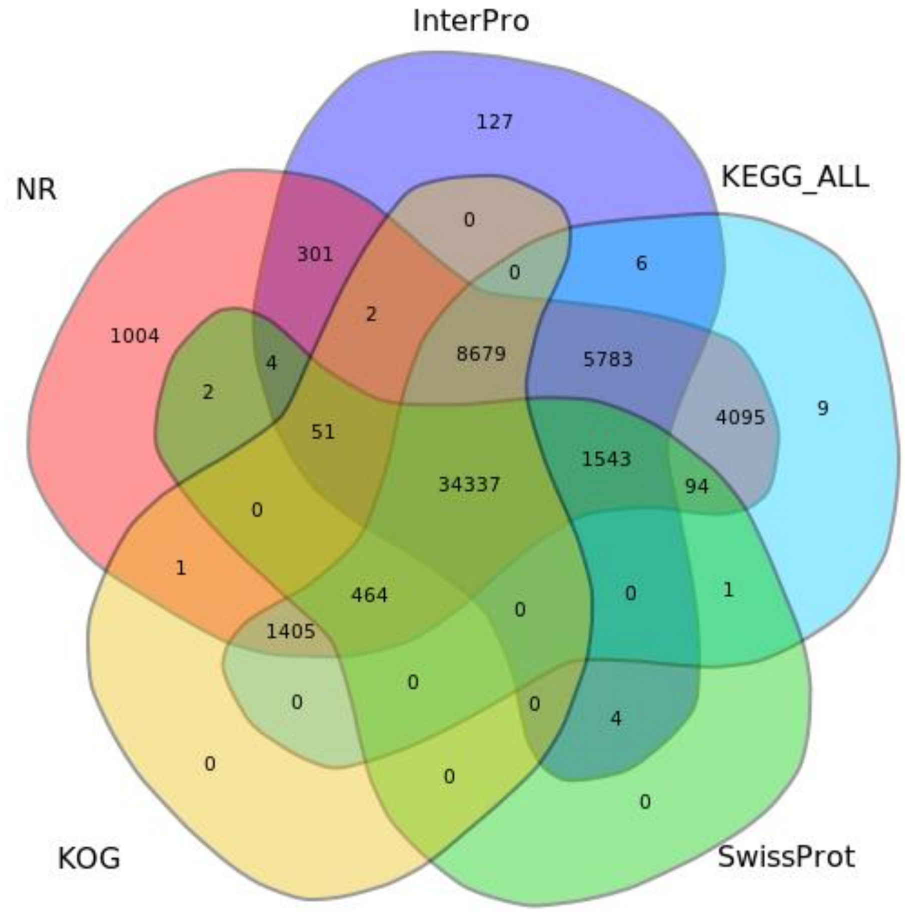

Figure S1 InterPro/GO/KEGG_KO/Swiss-Prot/NR Annotation Results Venn Diagram

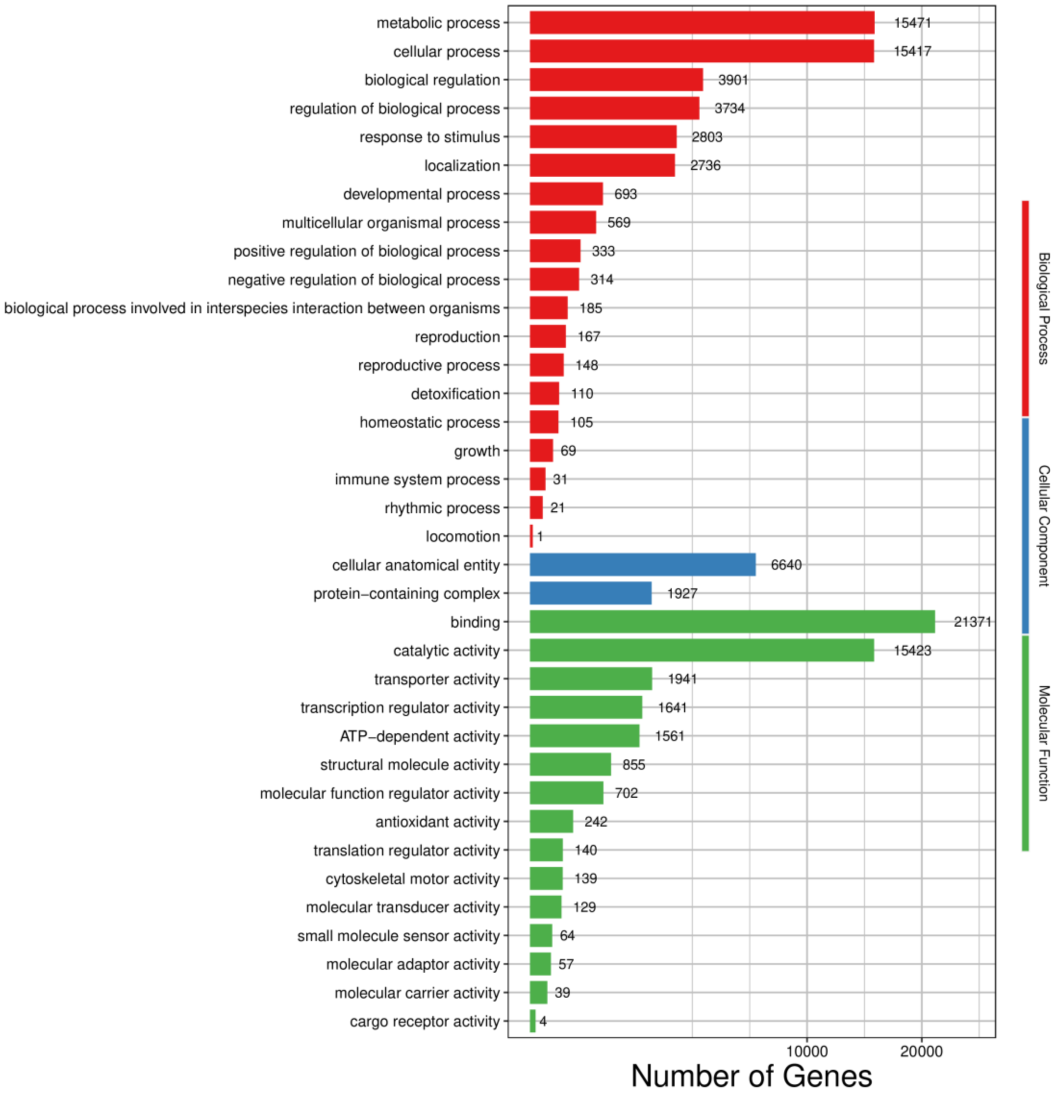

Figure S2 GO Category Statistics Bar Chart

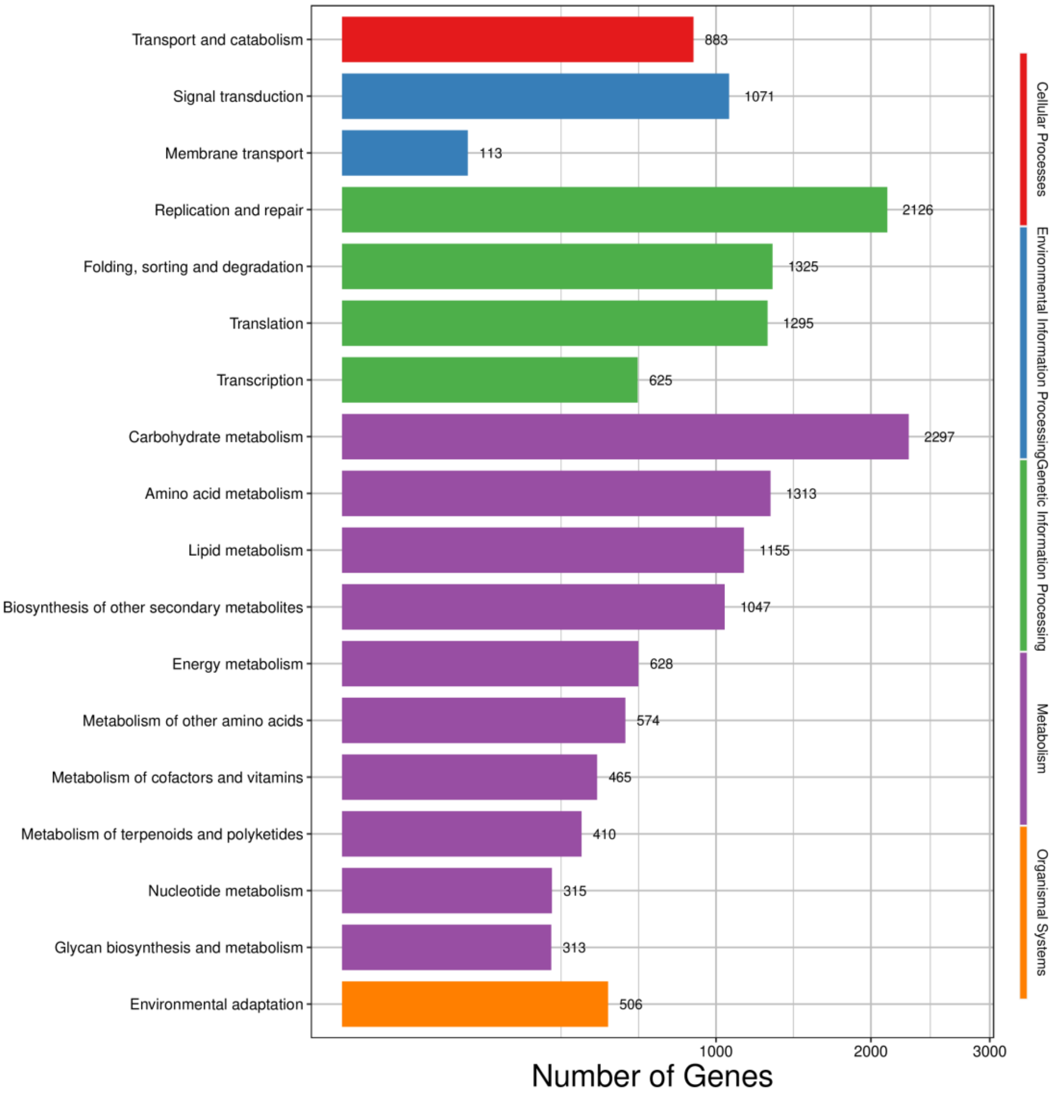

Figure S3 KEGG Classification Statistics Bar Chart

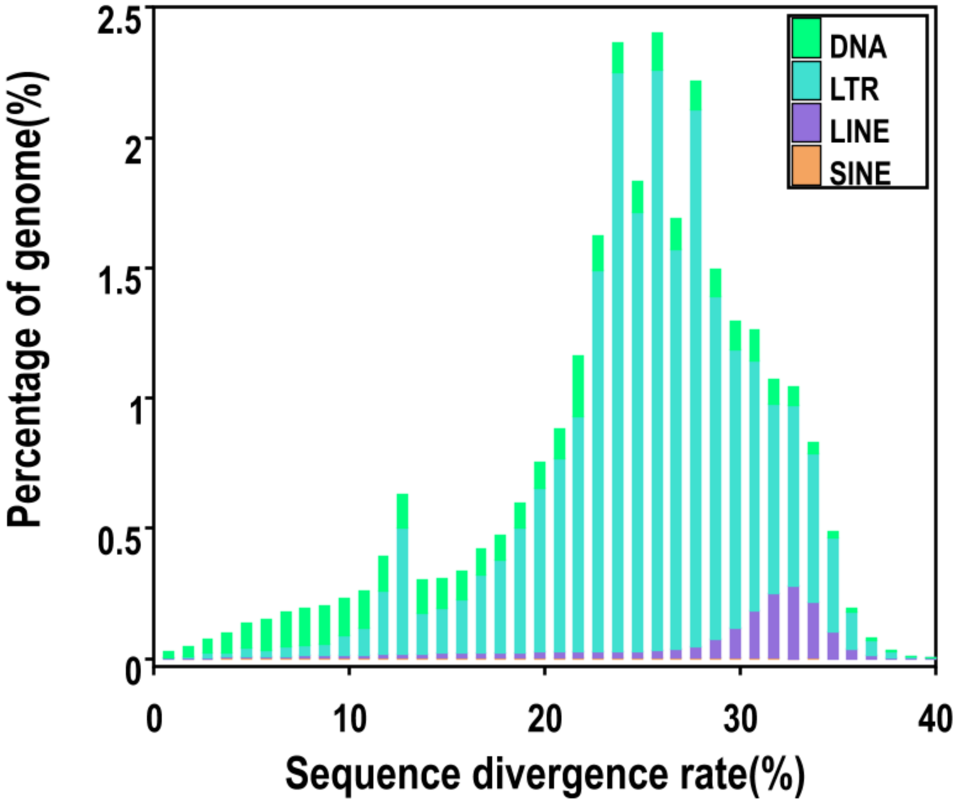

Figure S4 Distribution of Divergence Levels Among Four Transposable Element Sequences Annotated by RepeatMasker

Divergence distribution plot of TEs annotated using RepeatMasker with RepBase as the database. The x-axis represents the divergence between annotated TE sequences in the genome and their corresponding sequences in RepBase. The y-axis shows the percentage of TE sequences in the genome at each divergence level. Different TEs are color-coded for distinction.

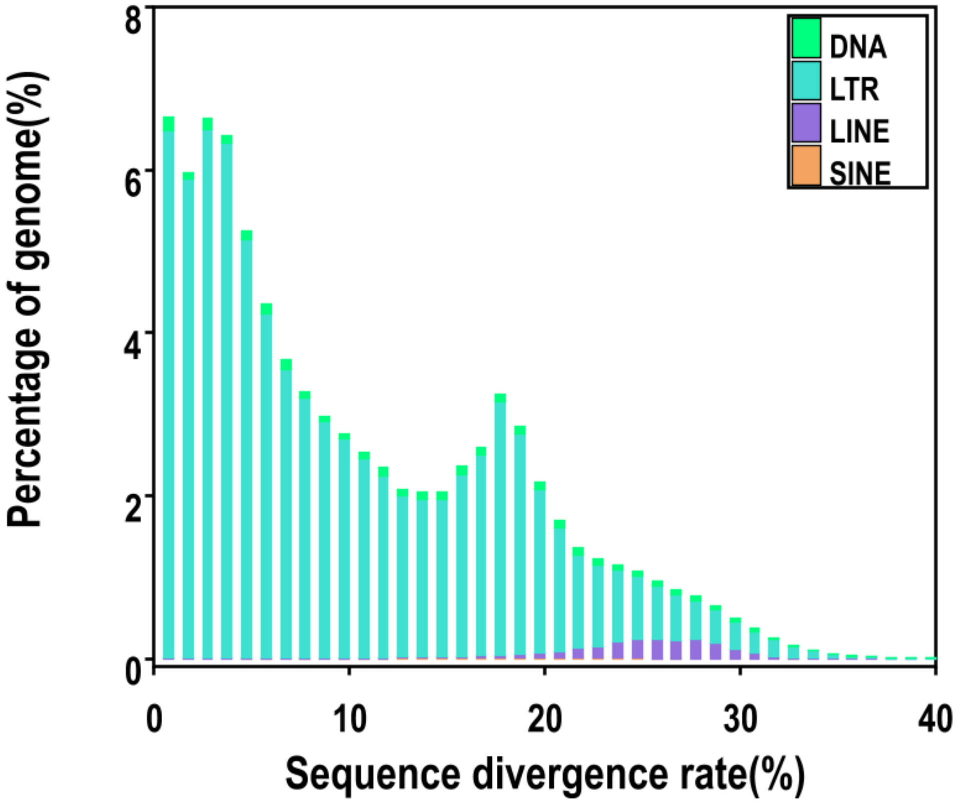

Figure S5 Distribution of divergence levels among four types of transposable element sequences predicted by de novo methods

The divergence distribution plot of TEs predicted by the de novo method shows the divergence between annotated TE sequences and their corresponding repetitive sequences in the de novo database on the x-axis, and the percentage of TE sequences at that divergence level relative to the entire genome on the y-axis. Different TEs are represented by distinct colors.
